# Giant and highly anisotropic magnetocaloric effects in single crystals of disordered-perovskite RCr_0.5_Fe_0.5_O_3_ (R = Gd, Er)

**DOI:** 10.1038/s41598-023-34258-w

**Published:** 2023-05-02

**Authors:** Hyun Jun Shin, Jin Seok Kim, Ki Won Jeong, Jong Hyuk Kim, Nara Lee, Young Jai Choi

**Affiliations:** Department of Physics, Yonsei University, Seoul, 03722 Korea

**Keywords:** Magnetic properties and materials, Magnetic properties and materials

## Abstract

Magnetic anisotropy is crucial in examining suitable materials for magnetic functionalities because it affects their magnetic characteristics. In this study, disordered-perovskite RCr_0.5_Fe_0.5_O_3_ (R = Gd, Er) single crystals were synthesized and the influence of magnetic anisotropy and additional ordering of rare-earth moments on cryogenic magnetocaloric properties was investigated. Both GdCr_0.5_Fe_0.5_O_3_ (GCFO) and ErCr_0.5_Fe_0.5_O_3_ (ECFO) crystallize in an orthorhombic *Pbnm* structure with randomly distributed Cr^3+^ and Fe^3+^ ions. In GCFO, the long-range order of Gd^3+^ moments emerges at a temperature of *T*_Gd_ (the ordering temperature of Gd^3+^ moments) = 12 K. The relatively isotropic nature of large Gd^3+^ moment originating from zero orbital angular momentum exhibits giant and virtually isotropic magnetocaloric effect (MCE), with a maximum magnetic entropy change of $\Delta {S}_{M}$ ≈ 50.0 J/kg·K. In ECFO, the highly anisotropic magnetizations result in a large rotating MCE characterized by a rotating magnetic entropy change $\Delta {S}_{\theta }$ = 20.8 J/kg·K. These results indicate that a detailed understanding of magnetically anisotropic characteristics is the key for exploring improved functional properties in disordered perovskite oxides.

## Introduction

Increased popularity of energy-efficient magnetic refrigeration in clean technology has inspired extensive research on novel magnetic materials to discover an effective technique of enhancing the magnetocaloric effect (MCE), which is described as the variation of temperature (*T*) in a magnetic material by applying the magnetic field (*H*)^1–4^. The MCE can be estimated by an adiabatic *T* change ($\Delta {T}_{\mathrm{ad}}$) and an isothermal magnetic entropy change ($\Delta {\mathrm{S}}_{\mathrm{M}}$) under the influence of *H*. Cryogenic magnetic refrigeration is crucial for obtaining sub-Kelvin temperatures as a substitute for^3^He/^4^He dilution refrigeration despite of increased cost and hydrogen gas liquefaction, which is utilized as an alternative fuel. Recently, large cryogenic MCE has been discovered in various insulating transition-metal oxides^5–7^ that possess easy manufacturability, chemical stability, and avoidance of refrigeration inefficiency because of eddy current. The beneficial aspect of MCE has been attained by the $\Delta {T}_{\mathrm{ad}}$ in various oxide magnets, such as Gd_2_CoMnO_6_^8^ ($\Delta {T}_{\mathrm{ad}}$ = 1.3 K for Δ*H* = 0–9 T at 2 K and $\Delta {T}_{\mathrm{ad}}$ = 8.3 K for Δ*H* = 0–9 T at 17 K ), SrFe_0.5_Co_0.5_O_3_^9^ ($\Delta {T}_{\mathrm{ad}}$ = 1.8 K for Δ*H* = 0–5 T at 330 K), HoMnO_3_^10^ ($\Delta {T}_{\mathrm{ad}}$ = 10.8 K for Δ*H* = 0–7 T at 11 K), CrO_2_^11^ ($\Delta {T}_{\mathrm{ad}}$ = 2.0 K for Δ*H* = 0–1.5 T at 390 K). Alternatively, the feasibility of magnetic refrigeration can be improved by developing a rotating MCE^8,12,13^, which can be achieved by rotating the refrigerant at constant *H.* The advantages of this method are technical simplicity and device compactness. However, strong magnetic anisotropy is essential for the realization of refrigerant cooling, which can be achieved using single-crystalline magnets whose intrinsic magnetocrystalline anisotropy originates from the anisotropic spin–orbit interaction that varies with the symmetry and structure. Cryogenic rotating MCE has been observed in several insulating oxide magnets, such as TbMnO_3_^14^ (the magnetic entropy change obtained by rotation, $\Delta {S}_{\theta }$ = 9.0 J/kg·K for 5 T at 15 K), HoMn_2_O_5_^15^ ($\Delta {S}_{\theta }$ = 12.4 J/kg·K for 7 T at 10 K), TmFeO_3_^16^ ($\Delta {S}_{\theta }$ = 9.0 J/kg·K for 5 T at 17 K), KTm(MoO_4_)_2_^17^ ($\Delta {S}_{\theta }$ = 9.8 J/kg·K for 5 T at 10 K), and KEr(MoO_4_)_2_^18^ ($\Delta {S}_{\theta }$ = 13 J/kg·K for 5 T at 10 K).

RCr_0.5_Fe_0.5_O_3_ (R = La, …, Lu) compounds crystallize in a disordered orthorhombic-perovskite structure with *Pbnm* space group having randomly distributed Cr^3+^ and Fe^3+^ ions owing to similar ionic radii of Cr^3+^ (0.615 Å) and Fe^3+^ (0.645 Å) ions^19–23^. The canted-antiferromagnetic order emerges in RFeO_3_ due to the dominant Fe^3+^-Fe^3+^ exchange couplings (*Γ*_4_(*G*_*x*_*A*_*y*_*F*_*z*_) in Bertaut’s notation)^24^. In RCr_0.5_Fe_0.5_O_3_, the *Γ*_4_ magnetic structure occurs at much lower *T* originating from a magnetic dilution effect of Cr^3+^ ions^28^. Extensive investigations on the series of compounds based on diverse magnetic phases and interactions reveal intriguing physical properties, such as metamagnetism^25,26^, exchange bias^27,28^, magnetodielectric effect^22,29,30^, and multiferroicity^31–34^. Additionally, large cryogenic MCEs in polycrystalline forms, such as GdCr_0.5_Fe_0.5_O_3_^20^ ($\Delta {S}_{M}$= 29.2 J/kg·K for *ΔH* = 0–4.5 T), Gd_2_NiMnO_6_^35^ ($\Delta {S}_{M}$= 37.2 J/kg·K for *ΔH* = 0–8 T), ErCr_0.5_Fe_0.5_O_3_^36^ ($\Delta {S}_{M}$= 12.4 J/kg·K for *ΔH* = 0–5 T), and DyCr_0.5_Fe_0.5_O_3_^33^ ($\Delta {S}_{M}$= 11.3 J/kg·K for *ΔH* = 0–4.5 T) have also been discovered. Various studies have hypothesized that large magnetic moments of magnetic rare-earth ions with strong anisotropy would significantly affect the cryogenic MCE. However, these studies focused only on polycrystalline specimens containing a large number of grains of all spatial orientations resulting in the average effect for observed physical properties.

To investigate the role of magnetic rare-earth ions and influence of anisotropic characteristics on MCE, single crystals of GdCr_0.5_Fe_0.5_O_3_ (GCFO) and ErCr_0.5_Fe_0.5_O_3_ (ECFO) were grown. For GCFO, large Gd^3+^ moments align below *T*_Gd_ (the ordering temperature of Gd^3+^ moments) = 12 K, which exhibits a relatively isotropic nature. The giant MCE is evidenced by the near-reversible magnetization along and perpendicular to the *c-*axis induces the maximum magnetic entropy change of $\Delta {S}_{M}$ = 49.8 and 48.8 J/kg·K, respectively. In contrast, Er^3+^ moments aligned along the *c*-axis below *T*_Er_ (the ordering temperature of Er^3+^ moments) = 11 K give rise to a highly anisotropic MCE. This generates a giant rotational MCE, i.e., $\Delta {S}_{\theta }$ = 20.8 J/kg·K. In view of the distinct magnetic aspects of disordered perovskites, these results contribute to the fundamental and applied research on magnetic materials.

## Results and discussion

**Figure 1:**
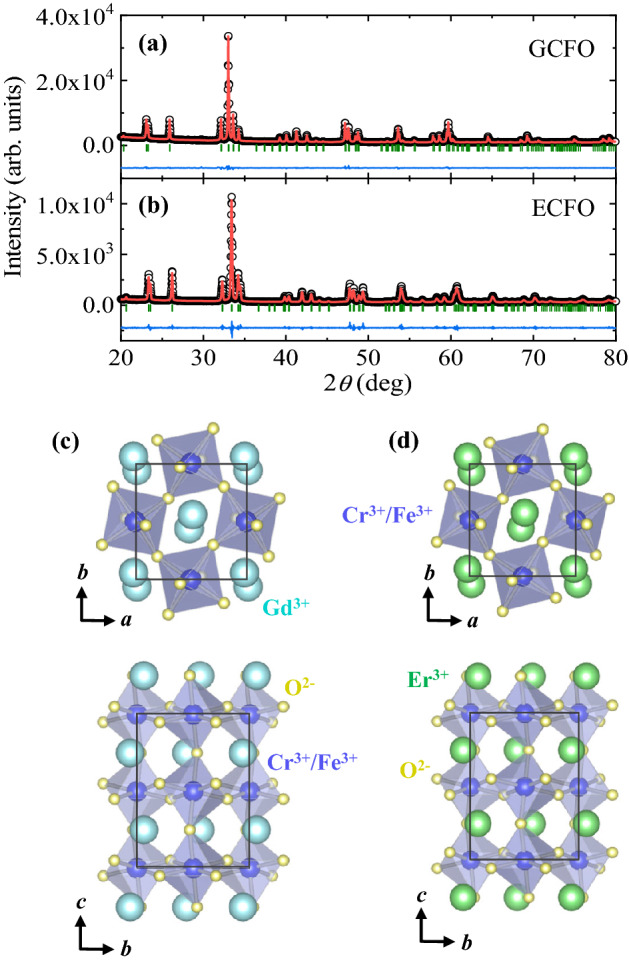
Crystallographic structures of GCFO and ECFO single crystals. (**a**, **b**) Observed (open circles) and calculated (solid line) powder X-ray diffraction patterns for the ground GdCr_0.5_Fe_0.5_O_3_ (GCFO) and ErCr_0.5_Fe_0.5_O_3_ (ECFO) single crystals. The short green lines denote the Bragg positions, and the blue curve indicates the difference between the observed and calculated patterns. (**c**) View of the crystallographic structure of GCFO from the *c*-axis and *a*-axis. The light blue, purple, and yellow spheres represent the Gd^3+^, Cr^3+^/Fe^3+^, and O^2−^ ions, respectively. The black box with the rectangular cross-sections depict a crystallographic unit cell. (**d**) View of the crystallographic structure of ECFO from the *c*-axis and *a*-axis. The green, purple, and yellow spheres represent the Er^3+^, Cr^3+^/Fe^3+^, and O^2-^ ions, respectively.

Figure 1a and 1b show the X-ray diffraction patterns measured at room temperature for the ground GCFO and ECFO, and the simulated patterns analyzed by the Rietveld refinement using the Fullprof software, respectively. The refined results indicate that GCFO and ECFO form an orthorhombic disordered perovskite with the *Pbnm* space group. The lattice constants were observed to be *a* = 5.3318 Å, *b* = 5.5674 Å, and *c* = 7.6379 Å for GCFO and *a* = 5.2411 Å, *b* = 5.5451 Å, and *c* = 7.5496 Å for ECFO. Additional details of crystallographic data are summarized in Table 1. The crystallographic structures of GCFO and ECFO viewed from the *c*- and *a*-axes are depicted in Fig. 1c and 1d, respectively. These structures are distinct from a double perovskite in which two different transition metal ions are alternately located in corner-shared octahedral units. Therefore, in disordered perovskites GCFO and ECFO, the sites of Cr^3+^ and Fe^3+^ ions present the randomly distributed arrangement arising from comparable ionic radii. The oxygen octahedral cages are considerably distorted because of the small radius of Gd^3+^/Er^3+^ ions, resulting in O^2-^ ion shifts in the bonds connecting the Cr^3+^/Fe^3+^ ions.

**Table 1:** Crystallographic data of GCFO and ECFO obtained from X-ray diffraction.

	GdCr_0.5_Fe_0.5_O_3_	ErCr_0.5_Fe_0.5_O_3_
Structure	Orthorhombic	Orthorhombic
Space group	*Pbnm*	*Pbnm*
*a* (Å)	5.3318	5.2411
*b* (Å)	5.5674	5.5451
*c* (Å)	7.6379	7.5496
*V* (Å^3^)	226.7254	219.4097
Gd /Er(x, y, z)	(0.98452, 0.06061, 0.25)	(0.98180, 0.06865, 0.25)
Cr (x, y, z)	(0.5, 0, 0)	(0.5, 0, 0)
Fe (x, y, z)	(0.5, 0, 0)	(0.5, 0, 0)
O_1_ (x, y, z)	(0.09541, 0.46746, 0.25)	(0.10199, 0.46368, 0.25)
O_2_ (x, y, z)	(0.70144, 0.29933, 0.05020)	(0.69014, 0.30857, 0.05442)
*R*_p_ (%)	9.74	16.1
*R*_wp_ (%)	7.99	14.7
*R*_exp_ (%)	6.70	9.52
*χ*^2^	1.42	2.38

**Figure 2:**
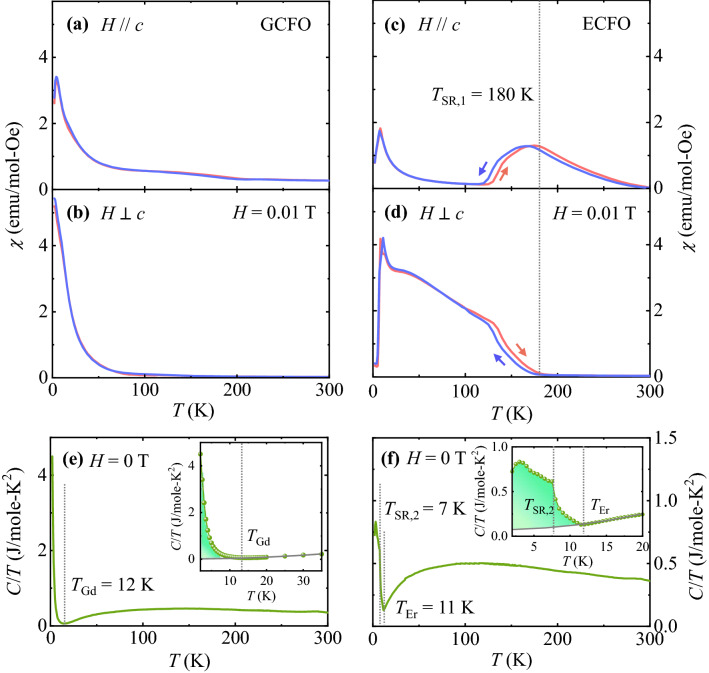
Magnetic susceptibility and heat capacity. (**a**, **b**) Temperature (*T*) dependence of magnetic susceptibility χ = *M*/*H* of single crystalline GCFO at *H* = 0.01 T measured on heating from 2 to 300 K after zero-field cooling (ZFC) and upon cooling at the same field (FC) parallel (*H//c*) and perpendicular (*H* $\perp$ *c*) to the *c*-axis. (**c**, **d**) *T* dependence of magnetic susceptibility χ = *M*/*H* of single crystalline ECFO at *H* = 0.01 T measured on heating from 2 to 300 K after ZFC and FC for *H//c* and *H* $\perp$ *c*. (**e**) Heat capacity divided by temperature (*C*/*T*) measured at *H* = 0 T and *T* = 2–300 K for GCFO. Inset shows *C*/*T* in the *T* = 2–35 K region. The gray curve was obtained by fitting, considering the influence of Cr^3+^/Fe^3+^ moments on *C*/*T* at a low-*T* regime. The vertical dashed line denotes the ordering *T* of Gd^3+^ moments as *T*_Gd_ = 12 K. The colored area indicates the contribution of Gd^3+^ ions to the magnetic entropy. (**f**)* C*/*T* measured at *H* = 0 T and *T* = 2–300 K for ECFO. Inset shows *C*/*T* in the *T* = 2–20 K region. The gray curve was obtained by fitting, considering the influence of Cr^3+^/Fe^3+^ moments on *C*/*T* at a low-*T* regime. The vertical dashed lines designate the ordering *T* of Er^3+^ moments as *T*_Er_ = 11 K and the 2nd spin-reorientation transition of Cr^3+^/Fe^3+^ moments, *T*_SR,2_ = 7 K. The colored area indicates the contribution of Er^3+^ ions to the magnetic entropy.

To examine the magnetic properties of GCFO and ECFO single crystals, the dependence of *T* on magnetic susceptibility $\chi =$ *M*/*H* was measured at $H$ = 0.01 T on warming after zero-field cooling (ZFC) and cooling in the same field (FC). The anisotropic $\chi$’s were obtained for the orientations that are parallel (*H//c*) and perpendicular to the *c*-axis (*H* $\perp$ *c*), as shown in Fig. 2a and b for GCFO and Fig. 2c and d for ECFO, respectively. Based on a previous study, the canted antiferromagnetic order of Fe^3+^ magnetic moments in GdFeO_3_ manifests at *T*_N_ = 661 K^37^. Unlike other orthoferrites, the canted moments along the *c*-axis do not rotate on further cooling. In GCFO, half of the Fe^3+^ ions are replaced by Cr^3+^ ions. However, the same tendency of canted moments aligned along the *c*-axis is sustained because $\chi$ for *H//c* appears to be larger than that for *H* $\perp$ *c* in the overall *T* range, except for the low-*T* regime (Fig. 2a and b). The Gd^3+^ moments in GCFO are antiferromagnetically ordered along magnetic easy-axis *c* in the low-*T* region, evidenced by the smaller magnitude and peaky feature of $\chi$ for *H//c*. Even without an orbital moment (*L* = 0 of Gd), Gd compounds still possess a very small magnetic anisotropy due to weak dipole–dipole interaction of the large Gd spin. This weak anisotropy is the reason why the Gd spins are pointing towards the *c*-axis, i.e., a particular easy-axis^38^.

**Figure 3:**
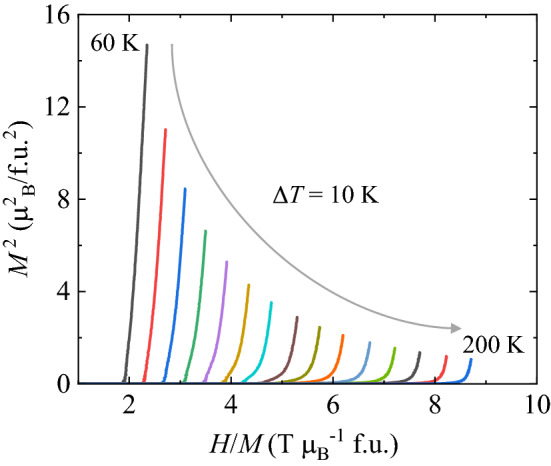
Belov-Arrott plot. Belov-Arrot plot for the ECFO crystal at *H//c* and *T* = 60—200 K.

In ErFeO_3_, the canted antiferromagnetic order of Fe^3+^ magnetic moments with a small net moment aligned along the *c*-axis occurs at *T*_N_ ≈ 640 K^39,40^ with *Γ*_4_ magnetic structure^41^. On further cooling, the net magnetic moment rotates to the *a*-axis by 90˚ at *T*_SR_ = 113 K by forming the *Γ*_2_(*F*_*x*_*C*_*y*_*G*_*z*_) magnetic structure, followed by the long range antiferromagnetic order of Er^3+^ magnetic moments aligned along the *c*-axis (*Γ*_1_(*C*_*z*_-type order)) at *T* = 3.4 K^42,43^. In ECFO, the *χ* properties of *H//*c and *H* $\perp$ c directions in the overall *T* range are strikingly different because of the strong anisotropic nature of the system (Fig. 2c and d). In contrast to the GCFO, the ECFO exhibits spin reorientation transition at *T*_SR,1_ ≈ 180 K, which indicates a considerable decrease in *χ* along the *c*-axis and an escalation of *χ* perpendicular to the *c*-axis. As shown in Fig. 3, we constructed the Belov-Arrott plot to determine the order of magnetic phase transition at *T*_SR,1_ ≈ 180 K. The slope was found to be positive for the overall regime of spin-reorientation, which suggests a second-order phase transition^44,45^. However, EFCO also exhibits signatures of a first-order phase transition such as thermally hysteretic behavior between ZFC and FC data (Fig. 2c) and the absence of a distinct peak in the specific heat (Fig. 2f). Thus, further studies are required to clearly identify the order of this spin-reorientation transition^46,47^. As *T* was further decreased, sharp anomalies were observed around 10 K indicating the antiferromagnetic order of Er^3+^ moments.

The *T* dependence of the heat capacity divided by *T* (*C/T*) measured at *H* = 0 T for GCFO exhibits a sharp increase below *T*_Gd_ = 12 K, indicating the ordering of Gd^3+^ moments, as shown in Fig. 2e. The influence of the ordering of magnetic Gd^3+^ moments on *C/T* in the low *T* regime was estimated by subtracting the contributions from Cr^3+^ and Fe^3+^ ions below *T*_Gd_. The subtracted part of *C/T* was obtained from the following equation:

1 $$C/T\sim \gamma + \rho T^{{1/2}} + \beta T^{2},$$

where $\gamma$, $\rho$, $\mathrm{and }\beta$ are coefficients corresponding to the electron, magnon, and phonon contributions of the Cr^3+^ and Fe^3+^ moments, respectively. Fitting the data to Eq. (1) resulted in the grey curve of *C/T* in the inset of Fig. 2e, which indicates the contribution from the interactions of Fe^3+^–Fe^3+^, Cr^3+^–Cr^3+^ and Cr^3+^–Fe^3+^ pairs and the interaction between the Gd^3+^ and Cr^3+^/Fe^3+^ sublattices at low *T*. The estimated entropy change based solely on the order of Gd^3+^ moments $\Delta$ *S*_Gd_ in zero *H* was observed to be 7.5 J/mole∙K. $\Delta$ *S*_Gd_ is 21.7% of the expected value of fully-saturated Gd^3+^ moments, i.e., $2R\mathrm{ln}(2J+1)$ = 34.6 J/mole∙K, where *R* is the gas constant and $J$ is the total angular momentum ($J$ = 7/2 for Gd^3+^ ions).

Previous experimental studies on neutron diffraction on the polycrystalline ECFO suggest that the spin configuration transforms from representation *Γ*_4_(G_x_A_y_F_z_) to *Γ*_2_(F_x_C_y_G_z_) on lowering *T* across *T*_SR,1_^26^. During the ordering of Er^3+^ moments at *T*_Er_ = 11 K, the C_z_ component belonging to *Γ*_1_ was observed on the Er^3+^ sublattice. Further decrease in *T* causes the 2nd spin-reorientation transition at *T*_SR,2_ = 7 K on the Cr^3+^/Fe^3+^ sublattice where the G_y_ component identified as another *Γ*_1_ phase_._ Across *T*_SR,2_, larger Er^3+^ moments were also observed. Furthermore, the measured *C/T* value reveals two different transitions, i.e., *T*_Er_ and *T*_SR,2_ at low-*T* regime, as shown in the inset of Fig. 2f. After subtracting the contribution of the Cr^3+^/Fe^3+^ sublattice represented by the gray curve, $\Delta$ *S*_Er_ in zero *H* was estimated to be 4.11 J/mole∙K, which is 8.9% of the expected value of the fully saturated Er^3+^ moments, $2R\mathrm{ln}\left(2J+1\right)$ = 46.1 J/mole∙K ($J$ = 15/2 for the Er^3+^ ions).

**Figure 4:**
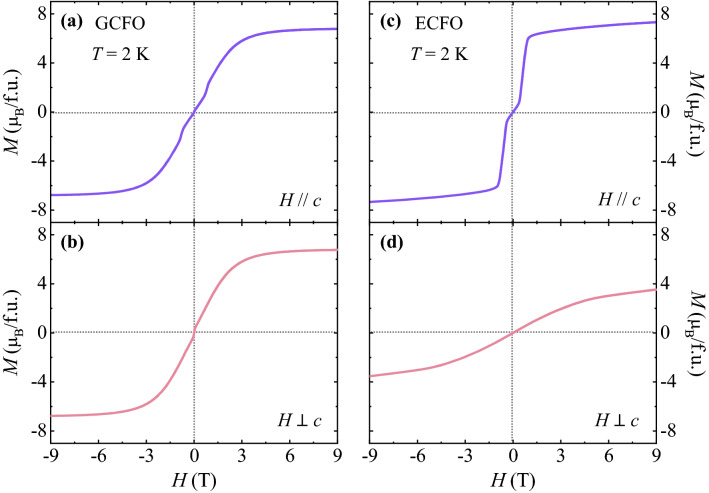
Isothermal magnetization. (**a**, **b**) Full magnetic hysteresis curve of the isothermal magnetization measured at 2 K up to *H* =  ± 9 T at *H//c* and *H* $\perp$ *c*, respectively, for GCFO. (**c**, **d**) Full magnetic hysteresis curve of the isothermal magnetization measured at 2 K up to *H* =  ± 9 T at *H//c* and *H* $\perp$ *c*, respectively, for GCFO.

As shown in Fig. 4, the magnetic anisotropies in GCFO and ECFO were investigated using isothermal *M* for two different orientations (*H//*c and *H* $\perp$ c) at 2 K, the former exhibiting an insignificant magnetic anisotropy. The initial *M* curve at *H//c* shows a weak magnetic transition at *H* = 0.85 T (Fig. 4a), suggesting a spin-flop transition because of the G_z_ component that is consistent with the transition observed in a previous study of the polycrystalline ECFO^26^. The value of *M* at a maximum *H* of 9 T is 6.75 _*μB*_/f.u. The consecutive sweeping of *H* reveals no hysteretic behavior with nearly zero remanent *M* and coercive field. The initial *M* curve at *H* $\perp$ *c* increases smoothly (Fig. 4b) and attains the same magnitude of *M* at 9 T as that at *H//c*. Contrarily, ECFO has a distinctive *H* dependence for each direction. The slope of the isothermal *M* curve for *H//c* is the greatest in the narrow *H* regime between 0.3 and 1.0 T; thereafter, it decreases and the *M* value is observed to be 7.32 ${\mu }_{\mathrm{B}}$/f.u. at 9 T. However, the initial *M* curve for *H* $\perp$ *c* varies smoothly and does not reach saturation at *H* up to 9 T. The *M* value at 9 T is found to be 3.58 ${\mu }_{\mathrm{B}}$/f.u., which is approximately half the value of *M* at 9 T for *H//c*.

In GCFO, same *M* values at the maximum *H* and similar shapes of *M* curves for different orientations imply the moderately isotropic nature of the Gd^3+^ moments associated with the half-filled 4f. electronic configuration (*S* = 7/2 and *L* = 0). Therefore, the crystal field effect affected by the symmetry of local environment can be minimum^48–50^. Contrarily, the Er^3+^ ion exhibits strong anisotropic properties in the ECFO system because its angular momentum (*L* = 6) breaks the local symmetry and the crystal field effect substantially affects the magnetocrystalline anisotropy^43,50^.

**Figure 5:**
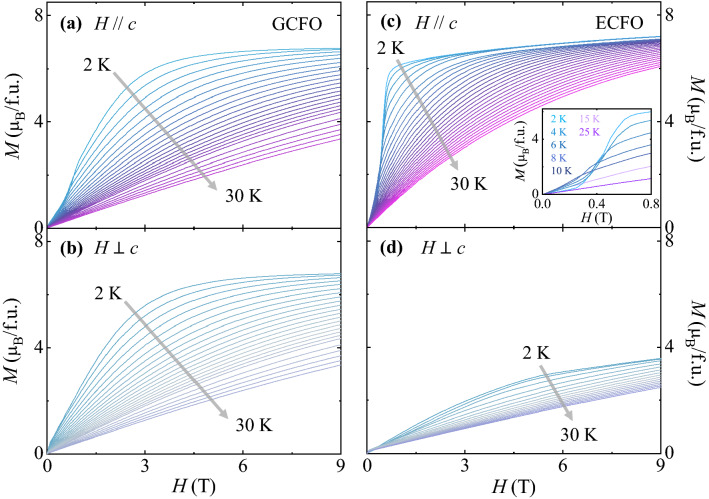
Initial curves of isothermal magnetization. (**a**, **b**) Initial curves of the isothermal magnetization for *H//c* and *H* $\perp$ *c*, respectively, at temperatures varying from 2 to 30 K for GCFO. (**c**, **d**) Initial curves of the isothermal magnetization for *H//c* and *H* $\perp$ *c*, respectively, at temperatures varying from 2 to 30 K for ECFO. Inset of c shows the initial curves of magnetization in the low-*H* region for *T* = 2, 4, 6, 8, 10, 15, and 25 K.

The contrasting magnetic properties of GCFO and ECFO lead to different MCE characteristics measured using the initial *M* curves with dense *T* steps for *T* = 2–30 K, as shown in Fig. 5. In GCFO, the almost isotropic magnetic properties resulted in the typical decreasing trend of the *M* values similarly for the two different orientations as *T* is increased (Fig. 5a and 5b). For *H*//*c* in ECFO, rapid increase in the initial *M* curve in the low-*H* regime at 2 K becomes broaden as *T* increases; hence, the *M* value at low *H* is lower than that at higher *T*, as plotted in the inset of Fig. 5c. This characteristic of the initial *M* curves varies above 10 K; thus, the *M* value exhibits a typical reduction in most of the *H* regime as *T* increases. At *H* $\perp$ *c*, owing to the smaller magnitude and smooth variation of *M* values, the overall magnitude of *M* is reduced marginally but continually in the entire regime of *H* as *T* increases (Fig. 5d).

**Figure 6:**
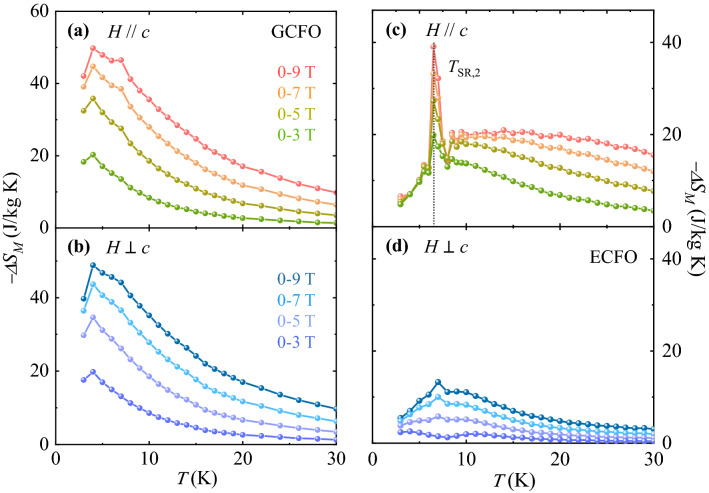
Anisotropic magnetocaloric effect in GCFO and ECFO crystals. (**a**, **b**) Anisotropic magnetocaloric effect in GCFO. *T* dependence of magnetic entropy change $-\Delta {S}_{M}$ for *H//c* and *H* $\perp$ *c* with *H* regimes of $\Delta H=$ 0–3, 0–5, 0–7, and 0–9 T, obtained by integrating the *T* gradient of the initial magnetization curves in Fig. 4a and b, respectively. (**c**, **d**) Anisotropic magnetocaloric effect in ECFO. *T* dependence of magnetic entropy change $-\Delta {S}_{M}$ for *H//c* and *H* $\perp$ *c* with *H* regimes of $\Delta H=$ 0–3, 0–5, 0–7, and 0–9 T, obtained by integrating the *T* gradient of the initial magnetization curves in Fig. 4c and d, respectively.

The MCE in GCFO and ECFO was quantified by calculating the isothermal magnetic entropy change, $\Delta {S}_{M}$, at a given *T* using the Maxwell’s relation:

2 $$\Delta {S}_{M}\left(T,H\right)={-\mu }_{0}{\int }_{0}^{{H}_{f}}\frac{\partial M\left(T,H\right)}{\partial T}dH,$$

where ${\mu }_{o}$ is the magnetic permeability of free space, *H*_*f*_ is the end point of *H* for the integral (*H*_*f*_ = 3, 5, 7, and 9 T), and the *T* gradient of *M*. $\frac{\partial M\left(T,H\right)}{\partial T}$ was estimated using the slope of two consecutive data points. The *T*-dependence of estimated $\Delta {S}_{M}(T)$ for *H//c* and *H* $\perp$ *c* is plotted in Fig. 6, for the *H* regimes of *ΔH* = 0–3, 0–5, 0–7, and 0–9 T respectively. The $\Delta {S}_{M}$ values for both orientations in GCFO present the largest at 4 K, where the maximum $\Delta {S}_{M}$ for *ΔH* = 0–9 T were achieved as 49.8 and 48.8 J/kg·K, respectively, for *H//c* and *H* $\perp$ *c* (Fig. 6a and b). This value of $\Delta {S}_{M}$ is greater than that of the other oxide materials, such as Dy_2_CoMnO_6_^51^ ($\Delta {S}_{M}$= 9.3 J/kg·K for *ΔH* = 0–7 T), HoMnO_3_^52^ ($\Delta {S}_{M}$= 13.1 J/kg·K for *ΔH* = 0–7 T), GdCrO_4_^53^ ($\Delta {S}_{M}$= 29.0 J/kg·K for *ΔH* = 0–9 T), and HoCrO_4_^54^ ($\Delta {S}_{M}$= 31.0 J/kg·K for *ΔH* = 0–8 T). Furthermore, non-hysteretic behavior of isothermal *M* indicates the absence of unnecessary $\Delta {S}_{M}$ loss.

**Figure 7:**
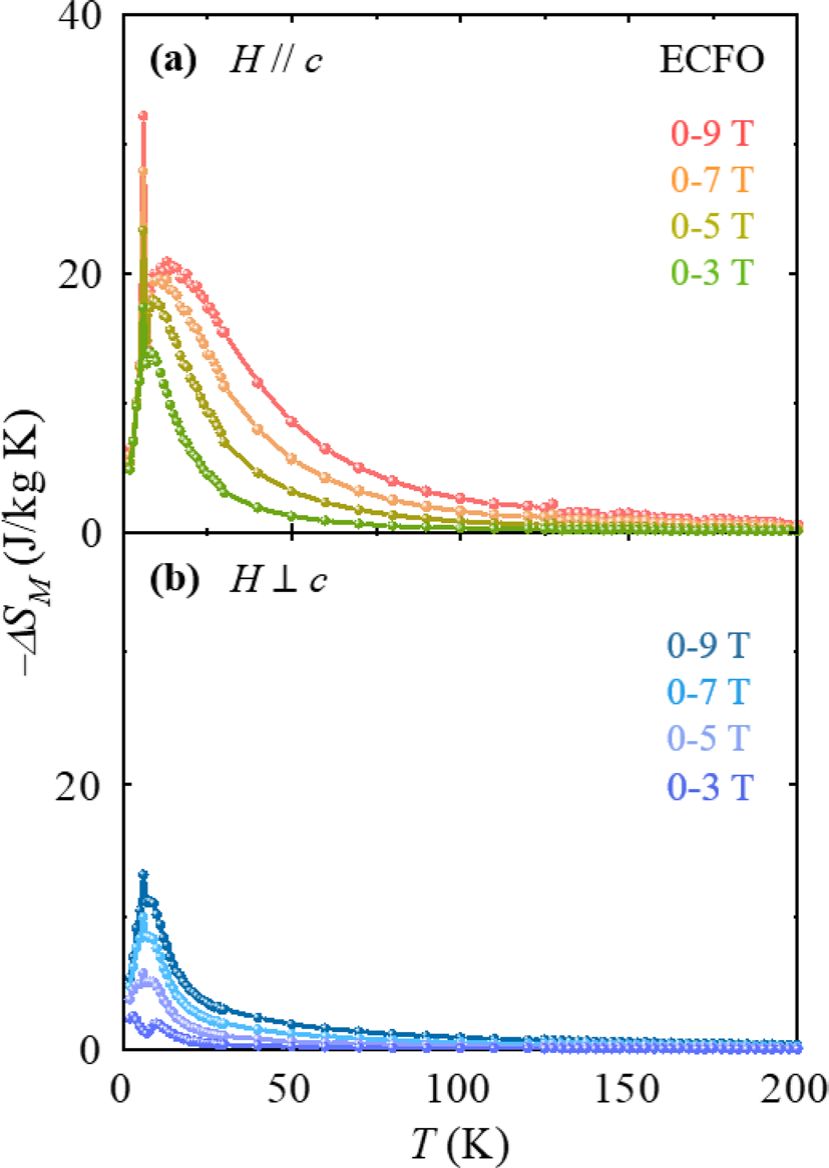
Anisotropic magnetic entropy change in ECFO. (**a**) *T* dependence of magnetic entropy change $-\Delta {S}_{M}$ for *H//c* with magnetic field regimes of $\Delta H=$ 0–3, 0–5, 0–7, and 0–9 T, respectively, obtained by integrating the *T* gradient of the initial magnetization curves in ECFO in the range *T* = 2–200 K. (**b**)* T* dependence of magnetic entropy change $-\Delta {S}_{M}$ for *H* $\perp$ *c* with magnetic field regimes of $\Delta H=$ 0–3, 0–5, 0–7, and 0–9 T, respectively, obtained by integrating the *T* gradient of the initial magnetization curves in ECFO in the range *T* = 2–200 K.

In ECFO, the peculiar anisotropy of $\Delta {S}_{M}$ was observed because the Er^3+^ spins mainly aligned along the *c*-axis. The intercrossed isothermal *M* values in the low-*H* regime for *H//c* (inset of Fig. 5c) considerably cancelled $\Delta {S}_{M}$; hence, $\Delta {S}_{M}$ for Δ*H* = 0–9 T was calculated to be 6.5 J/kg·K at 3 K (Fig. 6c). As *T* increases further, $\Delta {S}_{M}$ continues to increase and peaks sharply at *T*_SR,2_ with a maximum $\Delta {S}_{M}$ value of 39.1 J/kg·K. This feature was derived from the largest drop of isothermal *M* across *T*_SR,2_, which was verified by measuring initial *M* curves repeatedly in the low-*T* regime for various samples of ECFO crystals. Above *T*_SR,2_, $\Delta {S}_{M}$ shows a broad variation and its value is approximately 20 J/kg·K. In contrast with $\Delta {S}_{M}$ for *H//c*, the overall magnitude of $\Delta {S}_{M}$ for *H* $\perp$ *c* is largely reduced and its maximum value turns out to be 13.2 J/kg·K for Δ*H* = 0–9 T (Fig. 6d). Additionally, $\Delta {S}_{M}$ was estimated up to *T* = 200 K for investigating the influence of *χ* variation at *H* = 0.01 T across the spin-reorientation transition of *T*_SR,1_ (Figs. 2c and d). As shown in Fig. 7, magnitude and anisotropy of the estimated $\Delta {S}_{M}$ relevant to the spin-reorientation of Cr^3+^/Fe^3+^ moments were not pronounced. We also estimated relative cooling power (RCP) for both GCFO and ECFO crystals to show the potential of our single crystals as magnetic cryo-refrigerant. The RCP can be expressed by the following equation,

3 $${\text{RCP}} = \mathop \smallint \limits_{{T_{{{\text{cold}}}} }}^{{T_{{{\text{hot}}}} }} \Delta S_{M} \left( T \right)dT,$$

where *T*_cold_ = 2 K and *T*_hot_ was determined by the full width half maximum in $\Delta {S}_{M}$. Due to the rather isotropic nature of MCE, the RCP in the GCFO was estimated as 301 and 309 J/ kg at *H//c* and *H* $\perp$ *c*, respectively, for *ΔH* = 0–9 T. On the other hand, the RCP in the ECFO was found to be 623 J/kg at *H//c* and 169 J/kg at *H* $\perp$ *c*. The RCP has been estimated in polycrystalline specimens such as La_0.67_Sr_0.22_Ba_0.11_Mn_0.9_Fe_0.1_O_3_^55^ (241 J/kg for Δ*H* = 0–5 T), La_0.57_Mg_0.23_MnO_3_^56^ (176 J/kg for Δ*H* = 0–5 T), Ni_0.5_Zn_0.5_Fe_2_O_4_^57^ (161 J/kg for Δ*H* = 0–2.5 T) and La_0.5_Pr_0.2_Ca_0.1_Sr_0.2_MnO_3_^58^ (372 J/kg for Δ*H* = 0–5 T), and in single crystals such as La_0.7_Ca_0.3_MnO_3_^59^ (358 J/kg for Δ*H* = 0–5 T), h-DyMnO_3_^60^ (300 J/kg for Δ*H* = 0–5 T) and GdScO_3_^61^ (307 J/kg) for Δ*H* = 0–7 T).

**Figure 8:**
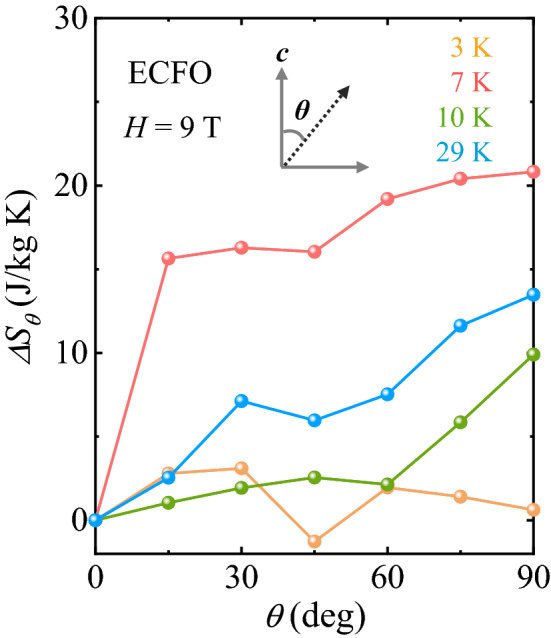
Rotating magnetocaloric effect in ECFO. Angular dependence of magnetic entropy change $\Delta {S}_{\theta }$ at *T* = 3, 7, 10, and 29 K with $\Delta H$ = 0–9 T, respectively. $\theta$ is the angle deviating from the *c*-axis, i.e., $\theta =0^\circ$ for *H//c* and $90^\circ$ for *H* $\perp$ *c*.

**Figure 9:**
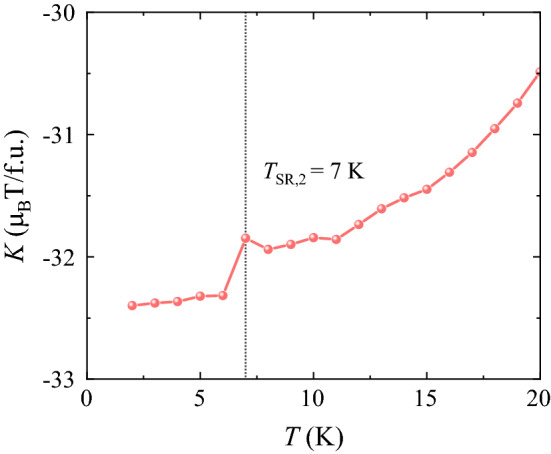
Magnetic anisotropy constant. Temperature dependence of magnetic anisotropy constant $K$.

To employ the conspicuous characteristics of the anisotropic MCE in disordered-perovskite ECFO, the rotating MCE was detected using the angle dependence of $\Delta {S}_{M}$, which is denoted by $\Delta {S}_{\theta }$, where $\theta$ is the angle deviated from the *c*-axis ($\theta$ = 0° for *H//c*, and $\theta$ = 90° for *H* $\perp$ *c*), as shown in the inset of Fig. 7. Figure 8 displays the resulting $\Delta {S}_{\theta }$ taken at *T* = 3, 7, 10 and 29 K for *H* = 9 T. The dissimilar *T* dependence of $\Delta {S}_{M}$ between *H//c* and *H* $\perp$ *c* in the low *T* regime (Fig. 6c and d) engenders the angle-dependent modulation of $\Delta {S}_{\theta }$, which changes significantly with *T*. $\Delta {S}_{\theta }$ at 3 K alters negligibly with *θ* rotation; however, $\Delta {S}_{\theta }$ at *T*_SR,2_ = 7 K shows a sudden increase to 15°. The continued variation in $\Delta {S}_{\theta }$ with *θ* yields a large rotational MCE demonstrated by the maximum change of $\Delta {S}_{\theta }$ = 20.8 J/kg·K, which would have applications in the rotary magnetic refrigerator technology. We have estimated the magnetic anisotropy constant for the ECFO crystal. The magnetic free energy of the ECFO crystal with uniaxial magnetocrystalline anisotropy can be described by $F=K{\mathrm{sin}}^{2}\theta -MH\mathrm{cos}\theta ,$ where the first term indicates the magnetic anisotropy energy with the angle *θ* deviating from the *c* axis and the second term denotes the Zeeman energy. The magnetic anisotropy constant $K$ was determined by the Sucksmith and Thompson method^62^, based on the experimental data for the ECFO crystal. The result shown in Fig. 9 exhibits a clear feature at *T*_SR,2_ = 7 K^63^. As *T* increases further, the gradual increase of $\Delta {S}_{\theta }$ with *θ* demonstrates a maximum $\Delta {S}_{\theta }$ of 9.9 and 13.5 J/kg·K at 10 and 29 K, respectively.

## Conclusion

This study investigated the anisotropic magnetic and magnetocaloric properties of disordered-perovskite GdCr_0.5_Fe_0.5_O_3_ and ErCr_0.5_Fe_0.5_O_3_. In GdCr_0.5_Fe_0.5_O_3_, the limited isotropic nature of Gd^3+^ moments owing to zero orbital angular momentum creates a weak directional dependence of giant magnetocaloric effect, characterized by maximum magnetic entropy changes of $\Delta {S}_{M}$ = 49.8 and 48.8 J/kg·K along and perpendicular to the *c*-axis, respectively. Non-magnetic hysteretic behavior of isothermal magnetization indicates the absence of dispensable loss of magnetocaloric effect. In contrast, ErCr_0.5_Fe_0.5_O_3_ reveals the sharpened feature of $\Delta {S}_{M}$ that is derived from the largest reduction of isothermal magnetization between adjacent measuring temperatures across the 2nd spin-reorientation along the *c*-axis at *T*_SR,2_ = 7 K. This particular anisotropy causes large rotary magnetocaloric effect with a maximum entropy change of $\Delta {S}_{\theta }$ = 20.8 J/kg·K. The results on different anisotropic magnetic properties of the disordered-perovskite compounds provide insights into suitable materials for magnetic functional applications.

## Methods

Single crystals of GCFO and ECFO were synthesized using conventional flux method with PbO, PbO_2_, and PbF_2_ fluxes in a high-*T* furnace. The stoichiometric ratios of Gd_2_O_3_/Er_2_O_3_, Cr_2_O_3_, and Fe_2_O_3_ powders for GCFO and ECFO were mixed and ground using a pestle in a corundum mortar. The mixture was pelletized and calcined at 1000 °C for 12 h. The calcined pellet was finely re-ground, pelletized, and sintered at 1200 °C for 12 h. The same procedure was repeated at 1250 °C for 24 h. The pre-sintered power containing fluxes was heated to 1260 °C in a platinum crucible for 24 h until it was completely dissolved. Thereafter, it was slowly cooled to 850 °C at the rate of 2 °C/h and further cooled to room temperature *T* at the rate of 100 °C/h. To identify the crystallographic structures of GCFO and ECFO crystals, X-ray diffraction was performed using an X-ray diffractometer (Ultima IV, Rigaku Corp., Japan) with Cu-${\mathrm{\rm K}}_{\mathrm{\alpha }}$ radiation.

The *T* and *H* dependences of DC magnetization (*M*) were obtained using a vibrating sample magnetometer at *T* = 2–300 K and *H* = -9–9 T in a physical properties measurement system (PPMS, Quantum Design, Inc., USA). The dependence of *T* on specific heat (*C*) was measured using the standard relaxation method and PPMS.

## Data Availability

The data that support the findings of this study are available from the corresponding authors upon reasonable request. The datasets associated with the crystallographic structures that are analyzed in this study are available in the Crystallography Open Database (COD) repository, #3,000,434 (https://www.crystallography.net/cod/information_card.php?id=3000434&CODSESSION=cbg4dlc90k4ciiuucsm3fkq2a3) and #3,000,435 (https://www.crystallography.net/cod/information_card.php?id=3000435&CODSESSION=cbg4dlc90k4ciiuucsm3fkq2a3).
